# Intra- and peritumoral radiomics nomogram based on DCE-MRI for the early prediction of pathological complete response to neoadjuvant chemotherapy in breast cancer

**DOI:** 10.3389/fonc.2025.1561599

**Published:** 2025-06-04

**Authors:** Yun Zhu, Shuni Zhang, Wei Wei, Li Yang, Lingling Wang, Ying Wang, Ye Fan, Haitao Sun, Zongyu Xie

**Affiliations:** ^1^ Department of Radiology, The First Affiliated Hospital of Bengbu Medical University, Bengbu, Anhui, China; ^2^ Department of Radiology, Anhui No.2 Provincial People’s Hospital, Hefei, China; ^3^ Department of Medical Imaging Diagnostics, Bengbu Medical University, Bengbu, China; ^4^ Department of Clinical Medicine, Bengbu Medical University, Bengbu, Anhui, China; ^5^ Zhongshan Hospital, Fudan University, Shanghai, China

**Keywords:** breast cancer, neoadjuvant chemotherapy, pathological complete response, intratumoral, peritumoral, radiomics, nomogram

## Abstract

**Purpose:**

This study aimed to create a nomogram model (NM) that combines clinical-radiological factors with radiomics features of both intra- and peritumoral regions extracted from pretherapy dynamic contrast-enhanced magnetic resonance imaging (DCE-MRI) images, in order to establish a reliable method for early prediction of pathological complete response (pCR) to neoadjuvant chemotherapy (NAC) in patients with breast cancer.

**Methods:**

A total of 214 patients were randomly divided into a training set (n=149) and a test set (n=65) in a ratio of 7:3. Radiomics features were extracted from intratumoral region and 2-mm, 4-mm, 6-mm, 8-mm peritumoral regions on DCE-MRI images, and selected the optimal peritumoral region. The intratumoral radiomics model (IRM), 2-mm, 4-mm, 6-mm, 8-mm peritumoral radiomics model (PRM), the combined intra- and the optimal peritumoral radiomics model (CIPRM) were constructed based on five machine learning algorithms, and then the radiomics scores (Rad-score) were obtained. Independent risk factors for clinical-radiological features were obtained by univariate and multivariate logistic regression analysis, and clinical model (CM) was constructed. Finally, the CIPRM Rad-score combined with clinical-radiological factors was used to construct a NM. The performance of different models were evaluated by receiver operating characteristic curve (ROC) analysis, calibration curve analysis, and decision curve analysis (DCA).

**Results:**

In our study, the 6-mm peritumoral size was considered to be the optimal peritumoral region. The CM is constructed based on three independent risk factors: estrogen receptor (ER), Ki-67, and breast edema score (BES). Incorporating ER, Ki-67, BES, and CIPRM Rad-score (combined intra- and 6-mm peritumoral) into the nomogram achieved a reliable predictive performance. And the area under the curve (AUC), sensitivity, specificity, and accuracy of the NM was 0.911, 0.848, 0.831, 0.826 for the training set and 0.897, 0.893, 0.784, 0.815 for the test set, respectively.

**Conclusion:**

The NM has a good value for early prediction of pCR after NAC in breast cancer patients.

## Introduction

Breast cancer has become one of the most common cancers worldwide and the leading cause of cancer death in women (1). According to “Cancer Statistics, 2024” published by the American Cancer Society, there are expected to be 310,720 new cases and 42,250 deaths from breast cancer in women in the United States in 2024, accounting for 32% and 15% of all new cases and deaths from cancer in women, respectively (2).Neoadjuvant chemotherapy (NAC) is a commonly used clinical treatment for early and locally advanced breast cancer (3). It can reduce the size of primary tumor and metastatic axillary lymph node (ALN), reduce the clinical stage, improve the surgical resection rate and breast preservation rate, obtain the body’s drug sensitivity reaction, and mediate the immune microenvironment (4, 5). The efficacy endpoints for NAC include complete loss of tumor cells, partial regression, no response, or tumor progression during treatment. Achieving pathologic complete response (pCR) after NAC prolongs overall survival and disease-free survival in breast cancer patients (6). Due to the complexity and high heterogeneity of breast cancer, some patients do not benefit from NAC, in addition, NAC drugs may also produce certain toxic side effects during treatment (7, 8). And the clinical need to adjust the treatment regimen in time to avoid delayed treatment due to disease progression. The pathological results after surgery are the gold standard to evaluate the efficacy of NAC, but there is a certain lag in time. Therefore, early and accurate prediction of the efficacy of NAC in breast cancer patients is of great significance for clinical development of individualized treatment and prognosis assessment.

Dynamic contrast-enhanced MRI (DCE-MRI) has high sensitivity in the diagnosis of breast cancer, which can reflect the way and degree of tumor enhancement, evaluate the hemodynamic characteristics of the tumor, and provide guidance for predicting the efficacy of NAC in breast cancer patients. However, it is easy to ignore the microscopic information of tumor when evaluating the efficacy of NAC solely by imaging features, and it is highly dependent on the technical level and subjective experience of the radiologist, resulting in low accuracy and specificity.

Radiomics is a new method of image analysis, which can extract the microscopic features that cannot be observed by the naked eye and conduct quantitative analysis and model construction, indirectly reflecting the heterogeneity and biological behavior of tumors (9–11). Radiomics has been applied to the diagnosis and differentiation of breast cancer, prediction of lymph node metastasis, molecular subtypes, histological grades, biomarkers and evaluation of NAC efficacy (12–16).

However, most of the previous studies focused on the intratumoral region and ignored the microscopic information contained in the peritumoral region. In recent years, some scholars have begun to study the value of peritumoral radiomics features in the diagnosis and treatment of breast cancer, and believe that the peritumoral radiomics features will affect the prediction performance of the model (17–20). However, there have been no studies on the optimal peritumoral region size for using DCE-MRI radiomics to predict NAC efficacy in breast cancer patients. In this study, we first aimed to identify the optimal peritumoral region for predicting pCR status after NAC in breast cancer patients. In addition, the intratumoral and optimal peritumoral radiomics features extracted from DCE-MRI images combined with clinical-radiological factors to build a nomogram model (NM). To investigate the value of NM in predicting pCR status after NAC in breast cancer patients.

## Materials and methods

### Patients

This study was approved by the ethics committee of our hospital (no. [2023] 441), and the subject’s informed consent was exempted. A retrospective analysis was performed on 360 patients who underwent breast MRI examination and NAC in our hospital from March 2019 to September 2023.

**Figure 1:**
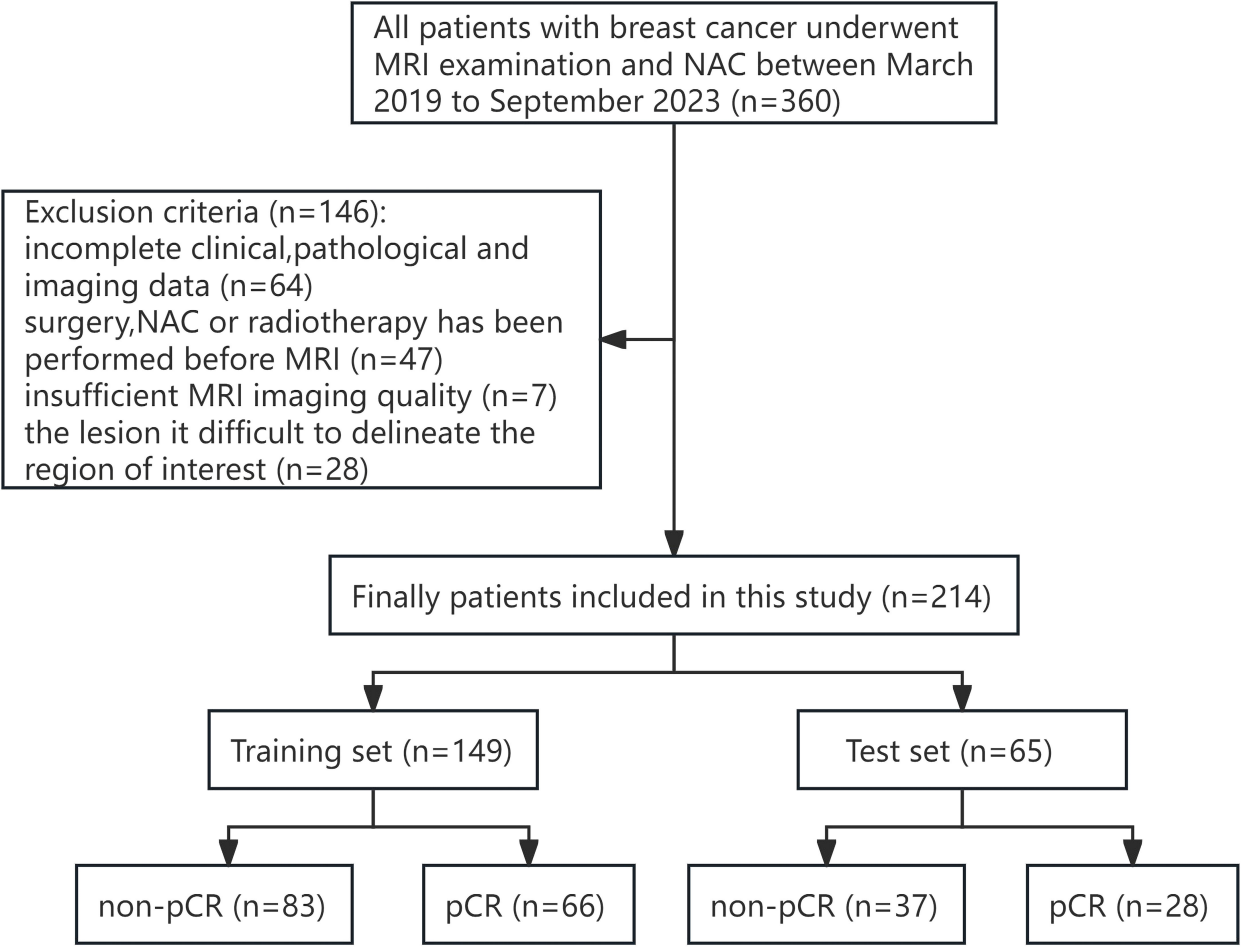
Workflow chart of the patients included in this study.

The inclusion criteria for patients were as follows: (1) The breast mass was confirmed by biopsy as primary invasive breast cancer; (2) NAC was performed after breast MRI examination; and (3) Surgical resection was performed after all courses of NAC. The exclusion criteria for patients were as follows: (1) Clinical, pathological and imaging data were incomplete; (2) Adjuvant therapy such as surgery or chemoradiotherapy was performed before MRI examination; (3) Poor MRI image quality affects the observation of lesions; and (4) The boundary of the lesion is not clear, and it is difficult to delineate the region of interest (ROI). Finally, 214 female patients who met the criteria of this study were included. The training set (n=149) and the test set (n=65) were randomly divided in a ratio of 7:3. **Figure 1** illustrates the patient selection workflow chart.

### MRI examination

All patients underwent MRI examinations were performed using a 3.0-T MRI scanner (Philips Achieva) with a dedicated breast coil (7-element SENSE breast coil).

All patients were placed in a prone position with both breasts naturally hanging down in the coil. The MRI acquisition sequence includes T1-weighted imaging (T1WI), fat-suppressed T2 weighted imaging (FS-T2WI), diffusion-weighted imaging (DWI), pre-contrast-enhancement T1WI, and DCE-MRI. Pre-contrast-enhancement T1WI was obtained before the contrast agent was injected. Then contrast agent GD-DTPA (Beilu Pharmaceutical, Beijing, China) was injected intravenously at a dose of 0.2 mmol/kg and at an injection rate of 2 ml/s, followed by 20 ml of normal saline at the same rate. A total of six phases of images were collected, and the collection time of each phase was 60s. Detailed MRI parameters are shown in **Supplementary Table S1**.

### Clinical-radiological features

Clinicopathological data included age, estrogen receptor (ER), progesterone receptor (PR), human epidermal growth factor receptor-2 (HER-2), Ki-67, molecular subtypes, neutrophil lymphocyte ratio (NLR), menopausal status, and NAC regimen. NAC treatment cycles and regimens are based on National Comprehensive Cancer Network (NCCN) guidelines (21), where patients typically receive 6-8 cycles of NAC, followed by surgical resection 2-3 weeks after completion of NAC. The NAC regimens mainly include three types: (1) taxane-based; (2) anthracycline-based, and (3) anthracycline-and taxane-based. In addition, patients with positive HER-2 may be treated with trastuzumab or pertuzumab.

By two radiologists (with 8 and 10 years of experience in breast radiology, respectively) who were blinded to clinicopathology of the patients, according to the 5th edition of the American College of Radiology’s Breast Imaging Reporting and Data System (BI-RADS), to analyze the characteristics of breast tumors on MRI images. In the event of disagreement, a third radiologist with 20 years of experience in breast radiology further analyzed the MRI images and made a final assessment.

The radiological features included: (1) background parenchymal enhancement (BPE); (2) tumor size (the maximum diameter of the tumor as observed on the DCE-T1WI image); (3) tumor shape; (4) tumor margin; (5) T2 intratumoral hyperintense; (6) lesion type, distinguished as mass or non-mass enhancement (NME); (7) time-signal intensity curve (TIC); (8) apparent diffusion coefficient (ADC); (9) breast edema score (BES), according to Harada et al. ‘s study (22), we evaluated breast edema on T2WI images and defined it as BES, which was divided into 1-4 points (BES1: no edema; BES2: peritumoral edema; BES3: prepectoral edema; BES4: subcutaneous edema) (10). short diameter of ALN; and (11) number of lesions. For patients with multiple lesions, only the largest lesions were evaluated for the study.

### Pathological evaluation

Immunohistochemical (IHC) were performed on specimens obtained after each patient’s needle biopsy to determine the status of ER, PR, HER-2, and Ki-67. When at least 1% of carcinoma nuclei show ER or PR positive staining, this indicates a positive status for ER or PR, respectively (23). The expression status of HER-2 can be determined according to the IHC score. An IHC score of 3+ is defined as a positive HER-2 status, an IHC score of 0 or 1+ is defined as a negative HER-2 status. An IHC score of 2+ requires further by fluorescence *in situ* hybridization (FISH) to confirm the diagnosis, a positive result from FISH testing is defined as a positive HER-2 status (24). When the proliferation index of Ki-67 is equal to or more than 14%, it is considered to be high expression, and when it is lower than 14%, it is considered to be low expression (25). According to the expression status of ER, PR, HER-2 and Ki-67, breast cancer patients were divided into four molecular subtypes: Luminal A (ER+/PR+, HER-2-, and low expression of Ki-67), Luminal B (ER+/PR+, high expression of Ki-67 and/or HER-2 +), HER-2 overexpression (ER-, PR- and HER-2 +), and Triple-negative (ER-, PR-, and HER-2 -) (26). The postoperative pathological specimens were evaluated using the Miller-Payne grading system (27): grade 1, no change or some alteration to individual tumor cells, but no reduction in overall cellularity; grade 2, a minor loss of tumor cells but overall high cellularity (up to 30% loss of cellularity); grade 3, within 30 to 90% reduction in cancer cellularity; grade 4, a marked disappearance of tumor cells such that only small clusters or widely dispersed individual tumor cells remained (more than 90% loss of cellularity); and grade 5, no malignant cells identifiable in sections from the site of the tumor, or ductal carcinoma *in situ* might be present. Patients with Miller-Payne grade 5 were categorized achieving a pCR, and patients with grades 1-4 as achieving a non-pCR.

### Image acquisition and segmentation

**Figure 2:**
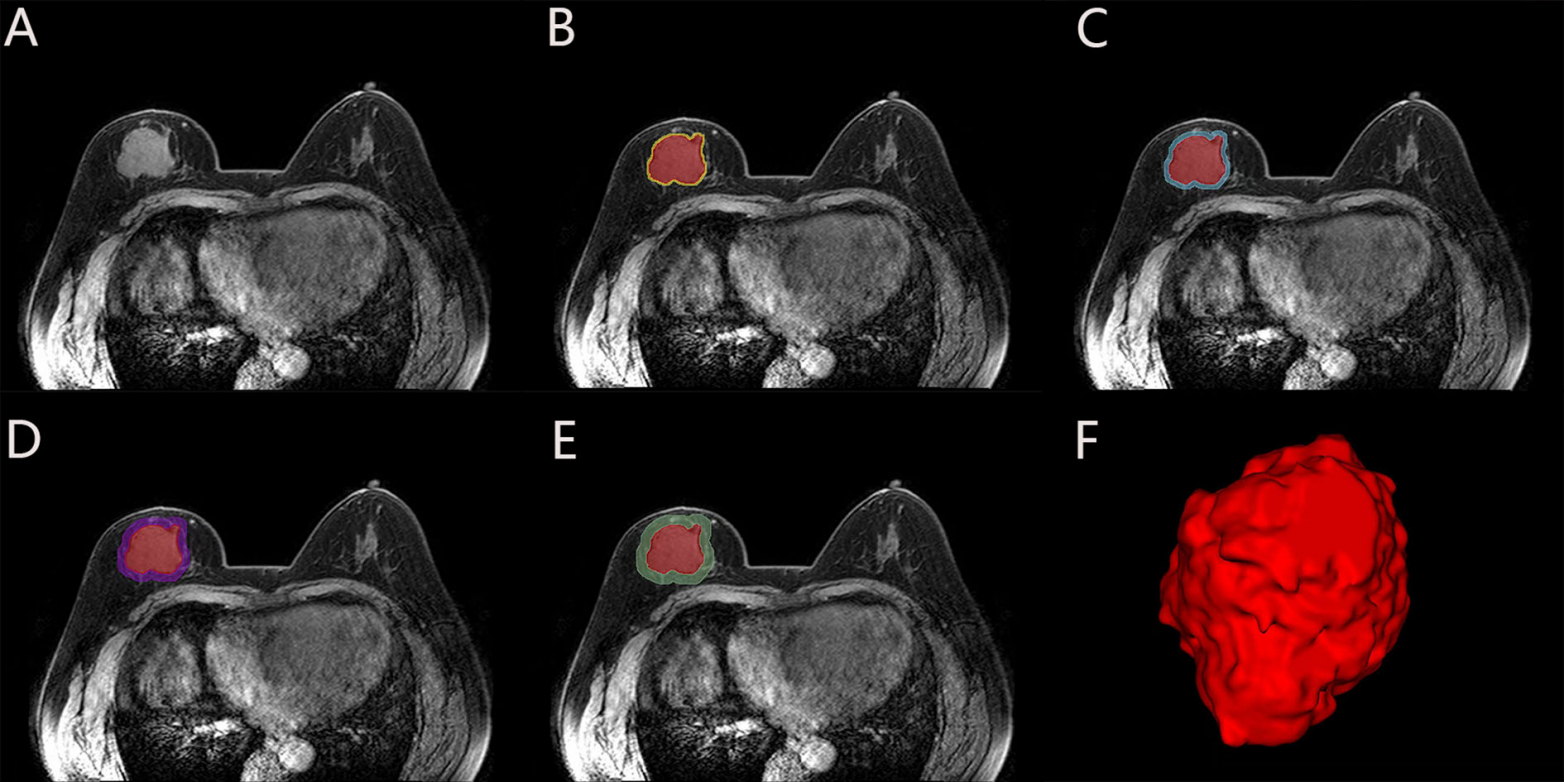
Schematic illustration of intratumoral and peritumoral ROI segmentation. The subject, a 49-year-old female diagnosed with invasive carcinoma in the right breast, exhibited non-pCR following NAC. **(A)** Maximal cross-sectional of tumor as observed on the second phase DCE-MRI image. **(B-E)** The red area is the intratumoral ROI, and the corresponding peritumoral ROIs were obtained by intratumoral ROI (red) automatic expansions by 2-mm (yellow), 4-mm (blue), 6-mm (purple) and 8-mm (green). **(F)** The 3D visualization intratumoral ROI with layer-by-layer delineation.

Since the contrast of signal intensity between breast cancer lesions and glandular background reaches its highest peak in the 60-120s after the injection of contrast agent, the lesion enhancement is the most obvious in this period, which contains more abundant tumor information. Therefore, we chose the second phase of the DCE-MRI sequence for the delineation of region of interest (ROI). Image segmentation was performed on the Darwin Intelligent Research Platform (Beijing Yizhun Medical AI, Beijing, China). The intratumoral ROI was segmented by two radiologists with 5 (radiologist 1) and 7 years (radiologist 2) of experience in breast radiology, respectively. They were blinded to the patients’ clinicopathological and radiological information. The two radiologists unified the delineation standard, and manually delineated layer by layer along the boundary of the lesion on the second phase of axial DCE-MRI images. Two weeks later, 10% of the patients were randomly selected and the segmentation process was repeated independently by radiologist 1 and radiologist 2, and the inter-/intraclass correlation coefficient (ICC) was calculated to evaluate the reproducibility of manual segmentation. The regions surrounding the tumor (i.e., 2-,4-,6- and 8-mm radius) were defined as the peritumoral regions in our study. The corresponding peritumoral ROIs were obtained by automatically expanding the intratumoral ROIs by 2-mm, 4-mm, 6-mm and 8-mm. Schematic illustration of intratumoral and peritumoral ROIs segmentation are shown in **Figure 2**. If the contour of the peritumoral region extends beyond the parenchyma of the breast, manual adjustments were made to the ROI to exclude these excess parts. Finally, the three dimensional (3D) ROIs of the intratumoral and 2-,4-,6- and 8-mm peritumoral regions were obtained.

### Radiomics feature extraction

The radiomics features of six different 3D-ROIs for each patient were extracted from the Darwin Intelligent research platform, including intratumoral, 2-, 4-, 6-, 8-mm peritumoral regions, intratumoral and optimal peritumoral regions. To further amplify the abundance of features, a variety of filters are used in our study, including exponential, logarithm, Laplacian of Gaussian (LoG), gradient, Local Binary Pattern 3D (LBP-3D), and wavelet filter. Finally, radiomics features were extracted from the original images and the images after various filters. The extracted radiomics features were normalized, and preprocessed to between (0,1) by using the maximum minimum value normalization method. The ICCs were calculated to quantify the consistency between the radiomics features extracted by two radiologists, retaining the radiomics features with ICCs > 0.75. Then we use the “Select K Best” method and select the f_calssif function to reduce the dimension of the features, and retaining the radiomics features with the sample variance F-values ranked in the top K%. Finally, using the least absolute shrinkage and selection operator (LASSO) logistic regression to reduce the dimensions of the remaining features, the optimal radiomics features for predicting pCR were finally selected.

### Model construction and validation

**Figure 3:**
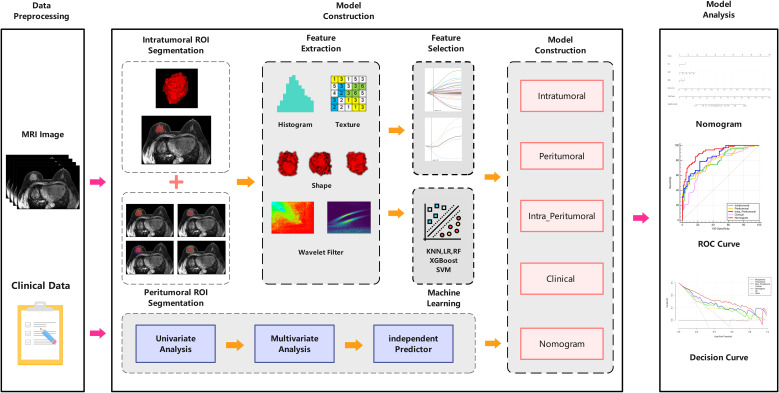
The workflow for the radiomics analysis.

The intratumoral radiomics model (IRM) and the 2-, 4-, 6- and 8-mm peritumoral radiomics model (PRM) were established based on five machine learning algorithms, and the corresponding radiomics score (Rad-score) was obtained according to the coefficient weighting of the selected optimal radiomics features in the respective models. Five machine learning algorithms include logistic regression (LR), random forest (RF), support vector machine (SVM), k nearest neighbors (KNN), and extreme gradient boosting (XGBoost), and compare the predictive performance of different machine learning algorithms. The area under the curve (AUC) of the receiver operating characteristic (ROC) was used to determine the optimal peritumoral region size for predicting pCR. Then based on the intratumoral and optimal peritumoral Rad-score, the combined intra- and peritumoral radiomics model (CIPRM) was established, and the confusion matrix was drawn to describe the performance of the model. In the training set, the clinical-radiological features highly correlated with pCR were selected as independent risk factors by uni- and multi-variate logistic regressions, and the clinical model (CM) was constructed. Finally, the CIPRM Rad-score combined with clinical-radiological factors was used to construct a nomogram model (NM).The ROC curves were drawn to evaluate the predictive performance of each model. DeLong test was used to compare the differences between the ROC curves of various models. The stability of the model was evaluated by tenfold cross-validation. The calibration curve and decision curve analysis (DCA) were used to evaluate the consistency and clinical practicability of the model. The workflow of radiomics analysis is shown in **Figure 3**.

### Statistical analysis

All statistical analyses were conducted using SPSS 26.0 software (SPSS, Chicago, IL), Medcalc software (Version 19.1.3), and R software (Version 4.4.1).

Kolmogorov-Smirnov was used to test the normality of the measurement data.The measurement data conforming to the normal distribution were represented with (mean ± standard deviation) and independent sample t-test was used. The measurement data that did not conform to normal distribution were represented by median (lower quartile-upper quartile) and Mann-Whitney U test was used. Counting data were expressed as frequency (constituent ratio), and using χ2 test, continuity correction test or Fisher exact probability method. Significant clinicopathological indicators and radiological features were screened by uni- and multi-variate logistic regressions. The ROC curves were plotted by Medcalc 19.1.3 software, and AUC, sensitivity, specificity and accuracy were calculated to evaluate the prediction performance of different models. Use R 4.4.1 software to draw nomogram, calibration curves and decision curves. When P < 0.05, the difference was considered statistically significant.

## Results

### Clinical-radiological features and construction of CM

In our study, 94 cases of the 214 patients achieved pCR, accounting for 43.9% (94/214), including 66 cases in the training set and 28 cases in the test set. 120 patients were non-pCR, accounting for 56.1% (120/214), including 83 cases in the training set and 37 cases in the test set. The clinical-radiological baseline features of breast cancer in the training and test sets are shown in **Table 1**.There were no significant differences in clinical-radiological features between the training set and the test set (p>0.05).

**Table 1:** Clinical-radiological baseline features of breast cancer in the training and test sets.

Characteristics	All	Training set (n=149)			All	Test set (n=65)			P-value
		non−pCR(n=83)	pCR(n=66)	P-value		non−pCR(n=37)	pCR(n=28)	P-value	
Age (years)	50.17 ± 10.57	50.51 ± 10.77	49.76 ± 10.37	0.669	51.94 ± 10.46	50.14 ± 12.28	54.32 ± 6.92	0.087	0.261
ER				0.003				0.002	0.269
Negative	81 (54.4)	36 (43.4)	45 (68.2)		30 (46.2)	11 (29.7)	19 (67.9)		
Positive	68 (45.6)	47 (56.6)	21 (31.8)		35 (53.8)	26 (70.3)	9 (32.1)		
PR				0.001				0.006	0.567
Negative	100 (67.1)	46 (55.4)	54 (81.8)		41 (63.1)	18 (48.6)	23 (82.1)		
Positive	49 (32.9)	37 (44.6)	12 (18.2)		24 (36.9)	19 (51.4)	5 (17.9)		
HER-2				0.037				0.040	0.425
Negative	73 (49.0)	47 (56.6)	26 (39.4)		28 (43.1)	20 (54.1)	8 (28.6)		
Positive	76 (51.0)	36 (43.4)	40 (60.6)		37 (56.9)	17 (45.9)	20 (71.4)		
Ki-67(%)	40.0 (22.5-60.0)	30.0 (20.0-50.0)	50.0 (37.5-60.0)	0.000	40.0 (30.0-60.0)	30.0 (22.5-60.0)	55.0 (40.0-70.0)	0.043	0.210
Molecular subtype				0.023				0.194	0.082
Luminal A	20 (13.4)	16 (19.3)	4 (6.1)		5 (7.7)	4 (10.8)	1 (3.6)		
Luminal B	52 (34.9)	32 (38.6)	20 (30.3)		28 (43.1)	19 (51.4)	9 (32.1)		
HER-2 overexpression	46 (30.9)	19 (22.9)	27 (40.9)		26 (40.0)	12 (32.4)	14 (50.0)		
Triple negative	31 (20.8)	16 (19.3)	15 (22.7)		6 (9.2)	2 (5.4)	4 (14.3)		
NLR	1.77 (1.35-2.17)	1.70 (1.33-2.10)	1.86 (1.39-2.21)	0.164	1.81 (1.45-2.60)	1.83 (1.42-2.70)	1.79 (1.50-2.50)	0.791	0.162
Menopausal Status				0.583				0.058	0.811
Premenopausal	76 (51.0)	44 (53.0)	32 (48.5)		32 (49.2)	22 (59.5)	10 (35.7)		
Postmenopausal	73 (49.0)	39 (47.0)	34 (51.5)		33 (50.8)	15 (40.5)	18 (64.3)		
NAC regimen				0.010				0.462	0.435
Taxane-based	3 (2.0)	1 (1.2)	2 (3.0)		3 (4.6)	1 (2.7)	2 (7.1)		
Anthracycline-based	57 (38.3)	40 (48.2)	17 (25.8)		21 (32.3)	14 (37.8)	7 (25.0)		
Anthracycline-and Taxane-based	89 (59.7)	42 (50.6)	47 (71.2)		41 (63.1)	22 (59.5)	19 (67.9)		
BPE				0.940				0.101	0.852
Minimal	29 (19.5)	16 (19.3)	13 (19.7)		14 (21.5)	5 (13.5)	9 (32.1)		
Mild	76 (51.0)	41 (49.4)	35 (53.0)		29 (44.6)	21 (56.8)	8 (28.6)		
Moderate	33 (22.1)	20 (24.1)	13 (19.7)		17 (26.2)	8 (21.6)	9 (32.1)		
Marked	11 (7.4)	6 (7.2)	5 (7.6)		5 (7.7)	3 (8.1)	2 (7.1)		
Tumor size (mm)	34.0 (26.0-45.0)	35.0 (28.0-47.0)	32.0 (23.0-42.3)	0.020	36.0 (27.5-51.0)	37.0 (28.0-51.0)	33.5 (25.5-47.5)	0.301	0.219
Shape				0.032				0.127	0.997
Regular	39 (26.2)	16 (19.3)	23 (34.8)		17 (26.2)	7 (18.9)	10 (35.7)		
Irregular	110 (73.8)	67 (80.7)	43 (65.2)		48 (73.8)	30 (81.1)	18 (64.3)		
Margin				0.076				0.950	0.810
Circumscribed	39 (26.2)	17 (20.5)	22 (33.3)		16 (24.6)	9 (24.3)	7 (25.0)		
Not circumscribed	110 (73.8)	66 (79.5)	44 (66.7)		49 (75.4)	28 (75.7)	21 (75.0)		
T2-intratumoral hyperintense				0.915				0.452	0.962
no	82 (55.0)	46 (55.4)	36 (54.5)		36 (55.4)	19 (51.4)	17 (60.7)		
yes	67 (45.0)	37 (44.6)	30 (45.5)		29 (44.6)	18 (48.6)	11 (39.3)		
Lesion type				0.018				0.209	0.992
Mass	126 (84.6)	65 (78.3)	61 (92.4)		55 (84.6)	29 (78.4)	26 (92.9)		
NME	23 (15.4)	18 (21.7)	5 (7.6)		10 (15.4)	8 (21.6)	2 (7.1)		
TIC				0.895				0.249	0.851
I	17 (11.4)	10 (12.0)	7 (10.6)		7 (10.8)	6 (16.2)	1 (3.6)		
II	38 (25.5)	22 (26.5)	16 (24.2)		19 (29.2)	11 (29.7)	8 (28.6)		
III	94 (63.1)	51 (61.4)	43 (65.2)		39 (60.0)	20 (54.1)	19 (67.9)		
ADC (*10^-3^mm^2^/s)	0.96 (0.77-1.04)	0.92 (0.74-1.02)	0.98 (0.78-1.05)	0.077	0.95 (0.83-1.11)	0.90 (0.80-1.11)	1.01 (0.87-1.17)	0.107	0.123
BES				0.024				0.044	0.144
1	30 (20.1)	13 (15.7)	17 (25.8)		19 (29.2)	7 (18.9)	12 (42.9)		
2	41 (27.5)	22 (26.5)	19 (28.8)		15 (23.1)	7 (18.9)	8 (28.6)		
3	35 (23.5)	16 (19.3)	19 (28.8)		8 (12.3)	7 (18.9)	1 (3.6)		
4	43 (28.9)	32 (38.6)	11 (16.7)		23 (35.4)	16 (43.2)	7 (25.0)		
Short diameter of ALN				0.175				0.138	0.405
<10mm	25 (16.8)	17 (20.5)	8 (12.1)		8 (12.3)	7 (18.9)	1 (3.6)		
≥10mm	124 (83.2)	66 (79.5)	58 (87.9)		57 (87.7)	30 (81.1)	27 (96.4)		
Number of lesions				0.596				0.161	0.308
Single	112 (75.2)	61 (73.5)	51 (77.3)		53 (81.5)	28 (75.7)	25 (89.3)		
Multiple	37 (24.8)	22 (26.5)	15 (22.7)		12 (18.5)	9 (24.3)	3 (10.7)		

pCR, pathological complete response; ER, estrogen receptor; PR, progesterone receptor; HER-2, human epidermal growth factor receptor-2; NLR, neutrophil lymphocyte ratio; NAC, neoadjuvant chemotherapy; BPE, background parenchymal enhancement; NME, non-mass enhancement; TIC, time-signal intensity curve; ADC, apparent diffusion coefficient; BES, breast edema score; ALN, axillary lymph node.

The univariate logistic regression analysis was performed on the data in the training set, and there were statistically significant differences in ER, PR, HER-2, Ki-67, molecular subtype, NAC regimen, tumor size, shape, lesion type and BES between non-pCR and pCR groups (p<0.05). The multivariate logistic regression analysis showed that ER (95% confidence interval [CI]:0.003-0.804; P=0.035), Ki-67 (95%CI: 1.011-1.059; P=0.004) and BES (P=0.043) were independent risk factors for pCR status, as shown in **Table 2** and **Supplementary Figure S1**. These three independent predictors were used to construct a CM.

**Table 2:** Uni- and multivariate logistic regression analysis of pCR status in breast cancer in the training set.

Characteristics		Univariate analysis		Multivariate analysis	
		OR (95% CI)	P-value	OR (95% CI)	P-value
Age		0.993 (0.963-1.024)	0.667		
ER	Negative	Reference			
	Positive	0.357 (0.182-0.703)	0.003	0.049 (0.003-0.804)	0.035
PR	Negative	Reference			
	Positive	0.276 (0.129-0.591)	0.001	0.631 (0.176-2.268)	0.481
HER-2	Negative	Reference			
	Positive	2.009 (1.041-3.876)	0.038	1.968 (0.337-11.495)	0.452
Ki-67(%)		1.035 (1.017-1.053)	0.000	1.035 (1.011-1.059)	0.004
Molecular subtype			0.030		0.344
	Luminal A	Reference			
	Luminal B	2.500 (0.731-8.552)	0.144	1.996 (0.215-18.541)	0.544
	HER-2 overexpression	5.684 (1.640-19.700)	0.006	0.166 (0.007-3.688)	0.256
	Triple negative	3.750 (1.019-13.795)	0.047	0.133 (0.008-2.262)	0.163
NLR		1.099 (0.771-1.565)	0.602		
Menopausal Status	Premenopausal	Reference			
	Postmenopausal	1.199 (0.628-2.290)	0.583		
NAC regimen			0.020		0.213
	Taxane-based	Reference			
	Anthracycline-based	0.213 (0.018-2.504)	0.218	0.083 (0.005-1.338)	0.079
	Anthracycline-and Taxane-based	0.560 (0.049-6.396)	0.640	0.113 (0.006-1.961)	0.134
BPE			0.935		
	Minimal	Reference			
	Mild	1.051 (0.445-2.482)	0.910		
	Moderate	0.800 (0.291-2.200)	0.665		
	Marked	1.026 (0.254-4.136)	0.972		
Tumor size		0.973 (0.949-0.997)	0.029	0.988 (0.952-1.026)	0.538
Shape	Regular	Reference			
	Irregular	0.446 (0.212-0.940)	0.034	0.478 (0.185-1.234)	0.127
Margin	Circumscribed	Reference			
	Not circumscribed	0.515 (0.246-1.079)	0.079		
T2-intratumoral hyperintense	no	Reference			
	yes	1.036 (0.541-1.984)	0.915		
Lesion type	Mass	Reference			
	NME	0.296 (0.104-0.846)	0.023	0.687 (0.158-2.991)	0.617
TIC			0.896		
	I	Reference			
	II	1.039 (0.325-3.317)	0.949		
	III	1.204 (0.422-3.434)	0.728		
ADC		4.044 (0.736-22.232)	0.108		
BES			0.029		0.043
	1	Reference			
	2	0.660 (0.256-1.704)	0.391	0.384 (0.119-1.239)	0.109
	3	0.908 (0.340-2.424)	0.847	0.871 (0.230-3.298)	0.839
	4	0.263 (0.097-0.711)	0.009	0.213 (0.060-0.759)	0.017
Short diameter of ALN	<10mm	Reference			
	≥10mm	1.867 (0.751-4.646)	0.179		
Number of lesions	Single	Reference			
	Multiple	0.816 (0.384-1.734)	0.596		

pCR, pathological complete response; ER, estrogen receptor; PR, progesterone receptor; HER-2, human epidermal growth factor receptor-2; NLR, neutrophil lymphocyte ratio; NAC, neoadjuvant chemotherapy; BPE, background parenchymal enhancement; NME, non-mass enhancement; TIC, time-signal intensity curve; ADC, apparent diffusion coefficient; BES, breast edema score; ALN, axillary lymph node; OR, odds ratio; 95% CI, 95% confidence interval.

### Optimal peritumoral region

In this study, we compared the predictive performance of pCR status based on radiomics features of four different peritumoral regions (2-, 4-, 6-, and 8-mm) (**Supplementary Table S2**). The results showed that 6-mm PRM had the highest maximum AUC (0.794 and 0.779) in both the training set and the test set. Therefore, we selected 6-mm as the optimal peritumoral region to predict pCR status for subsequent studies.

### Radiomics feature selection and construction of radiomics models

A total of 1781, 1781 and 3562 radiomics features were extracted from each patient’s intratumoral region, 6-mm peritumoral region, and intratumoral + 6-mm peritumoral region, respectively. The features of ICCs > 0.75 were retained, and the features dimensionality were reduced by screening threshold percentage and f_classif function. Finally, after LASSO regression, a total of 10, 11 and 10 optimal radiomics features highly correlated with pCR status were obtained. The IRM, 6-mm PRM and CIPRM were constructed, and calculate the Rad-Score. **Supplementary Table S3** illustrates the radiomics features and their respective coefficients of the three radiomics models. Among the three radiomics models, CIPRM had a maximum AUC of 0.851 and 0.840 in the training and test set, respectively, and its predictive performance was better than IRM and 6-mm PRM (**Supplementary Table S2**). According to the confusion matrix results, the sensitivity, specificity, and accuracy of the CIPRM were 0.788, 0.771 and 0.779 in the training set, and 0.750, 0.757 and 0.754 in the test set, respectively (**Supplementary Figure S2**). The Rad-Score of CIPRM is as follows:

Rad-Score=+2.339×wavelet-LHH_gldm_LargeDependenceLowGrayLevelEmphasis+ 2.184×exponential_firstorder_Kurtosis+1.763×gradient_glszm_SmallAreaLowGrayLevelEmphasis+1.751×wavelet-HLL_glcm_ClusterTendency-1.520×wavelet-HHL_glcm_Idmn-1.439×wavelet-LHH_firstorder_Kurtosis+1.205×wavelet-HHH_glcm_MCC+1.112×wavelet-HLH_gldm_LargeDependenceHighGrayLevelEmphasis+0.702×logarithm_glcm_Imc2-0.631×wavelet-LLH_ngtdm_Busyness-1.834

The comparison of prediction performance of different machine learning algorithms is shown in **Supplementary Table S2**. In the CIPRM, the AUC of the five machine learning algorithms in the training set ranged from 0.755 to 0.851, and the AUC in the test set ranged from 0.704 to 0.840. Compared with other machine learning algorithms, SVM obtained the largest AUC values in both the training set (0.851) and the test set (0.840), and the difference of AUC values in these two datasets was small, indicating that SVM had the best and most stable performance.

### Development and validation of NM

**Figure 4:**
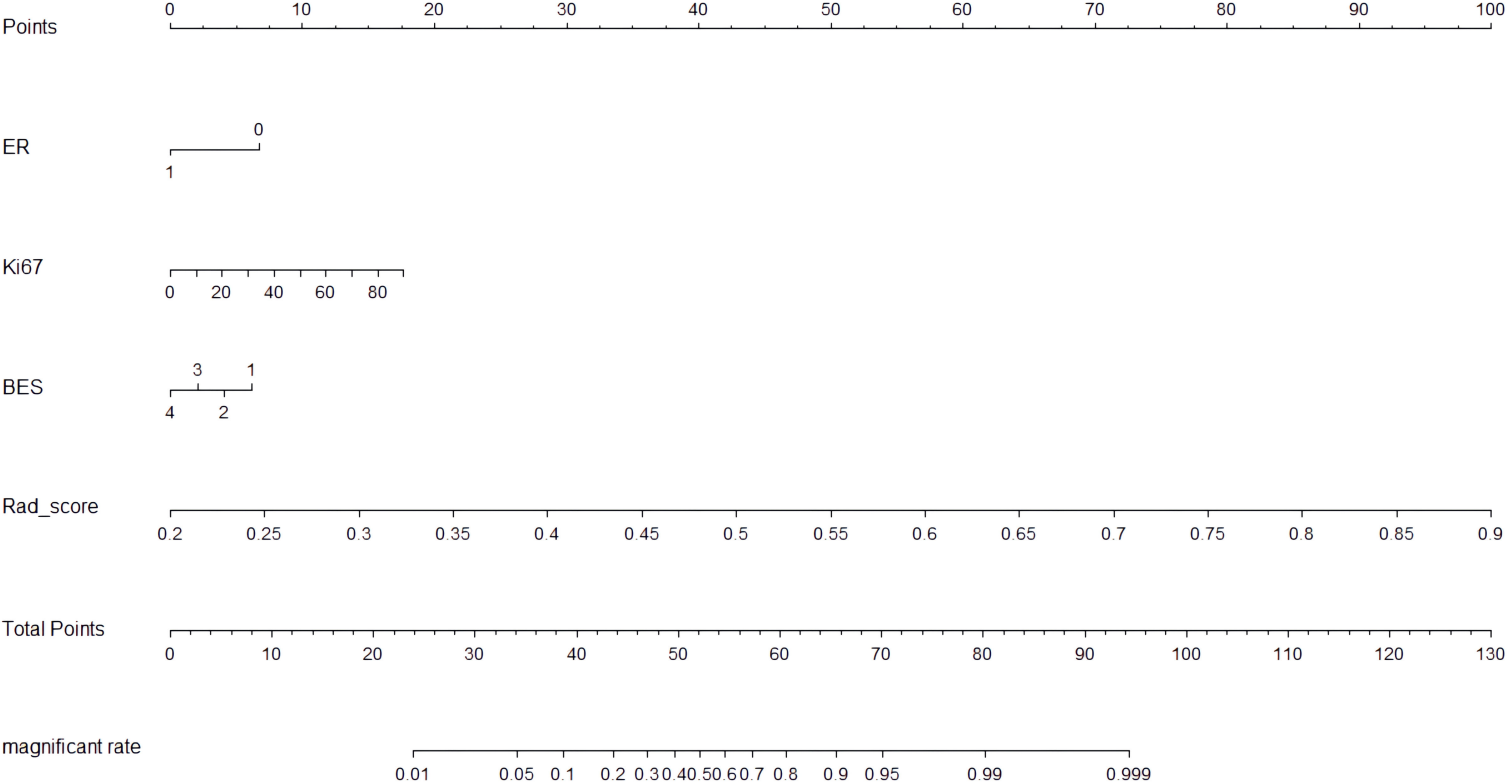
The nomogram was developed based on ER, Ki-67, BES and CIPRM Rad-score.

**Figure 5:**
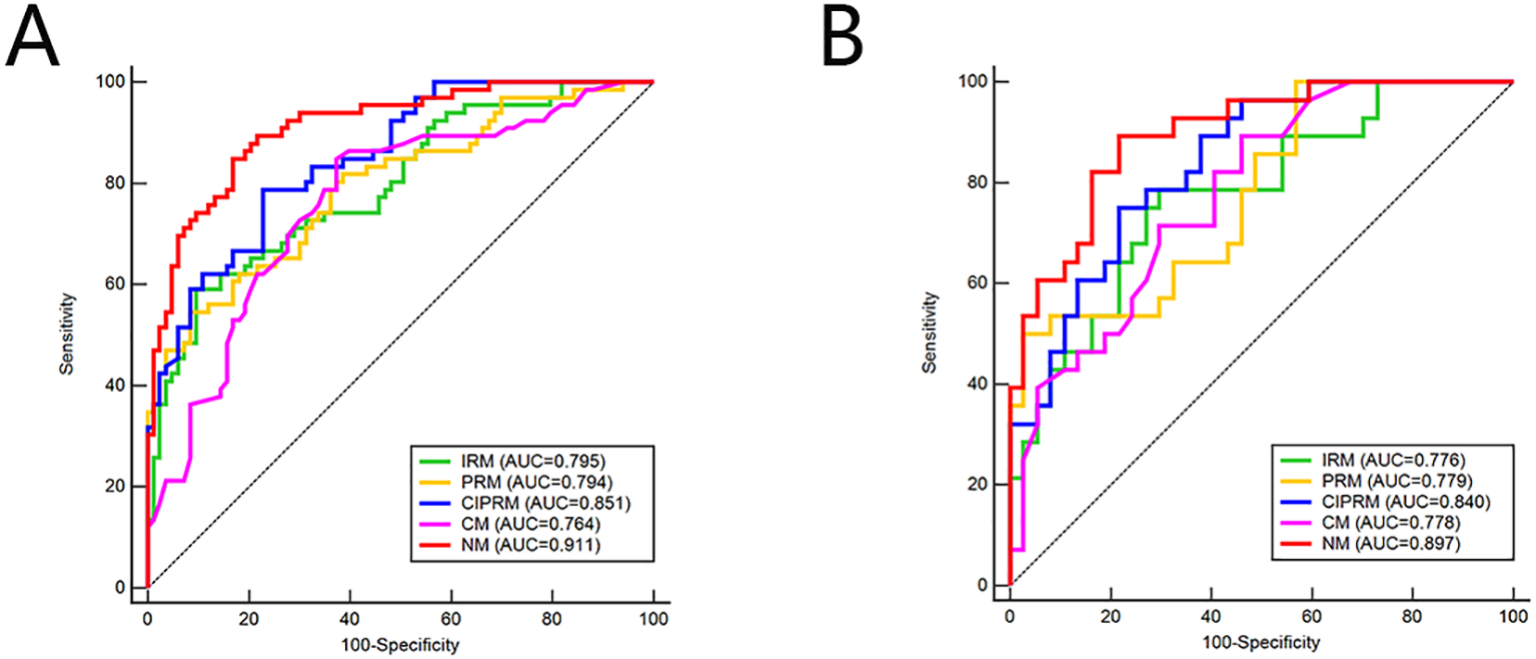
ROC curves of five models in the training set **(A)** and test sets **(B)**. IRM, intratumoral radiomics model; PRM, 6-mm peritumoral radiomics model; CIPRM, combined intra- and 6-mm peritumoral radiomics model; CM, clinical-radiological model; NM, nomogram model.

The CIPRM Rad-score combined with clinical-radiological factors (ER, Ki-67, and BES) was used to construct a NM. The nomogram shows that the CIPRM Rad-score has the greatest weight in the construction of the model, followed by Ki-67 (**Figure 4**). We finally constructed five models for predicting pCR states, including IRM, 6-mm PRM, CIPRM, CM and NM. **Figure 5** shows the ROC curves of these five models in the training set and the test set. ROC curve showed that the AUCs of the NM in the training set and the test set were 0.911and 0.897 respectively, which were higher than 0.795 and 0.776 for the IRM, 0.794 and 0.779 for the 6-mm PRM, 0.851 and 0.840 for the CIPRM, and 0.764 and 0.778 for the CM (**Figure 5**; **Table 3**).

**Table 3:** Performance of five models in the training and test sets.

Model	Group	AUC (95%CI)	Sensitivity	Specificity	Accuracy
IRM	Training set	0.795 (0.721-0.856)	0.591	0.904	0.738
	Test set	0.776 (0.656-0.870)	0.786	0.703	0.677
6-mm PRM	Training set	0.794 (0.721-0.856)	0.545	0.916	0.705
	Test set	0.779 (0.659-0.872)	0.50	0.973	0.662
CIPRM	Training set	0.851 (0.783-0.904)	0.788	0.771	0.779
	Test set	0.840 (0.728-0.919)	0.75	0.757	0.754
CM	Training set	0.764 (0.687-0.829)	0.847	0.627	0.705
	Test set	0.778 (0.657-0.871)	0.892	0.541	0.662
NM	Training set	0.911 (0.854-0.952)	0.848	0.831	0.826
	Test set	0.897 (0.796-0.958)	0.893	0.784	0.815

IRM, intratumoral radiomics model; PRM, peritumoral radiomics model; CIPRM, combined intra- and 6-mm peritumoral radiomics model; CM, clinical-radiological model; NM, nomogram model; AUC, area under the curve; 95% CI, 95% confidence interval.

**Figure 6:**
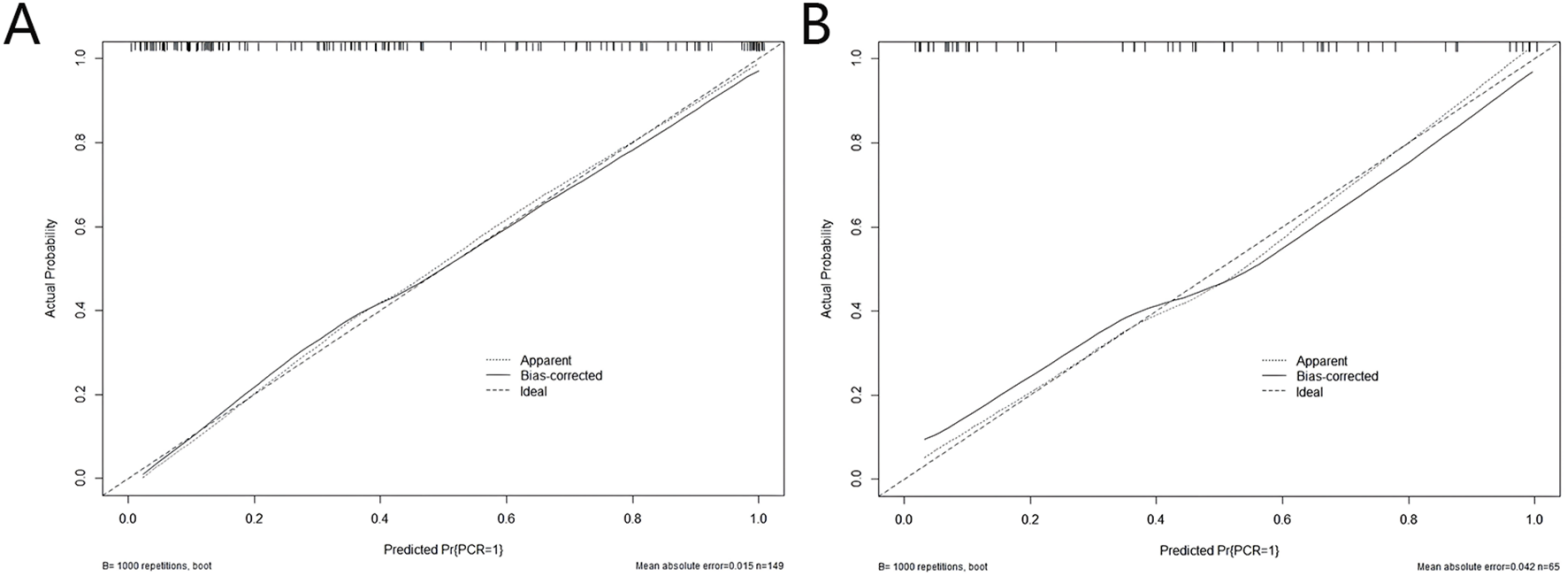
Calibration curves of the nomogram model in the training **(A)** and test sets **(B)**.

**Figure 7:**
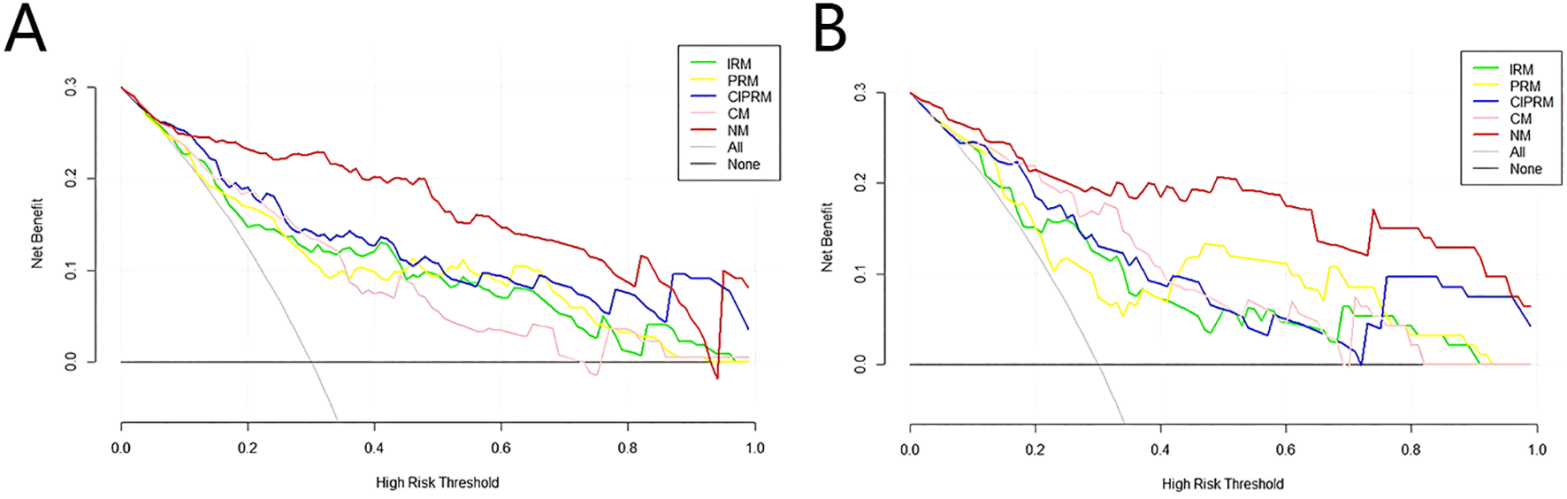
Decision curves of five models in the training set **(A)** and test sets **(B)**.IRM, intratumoral radiomics model; PRM, 6-mm peritumoral radiomics model; CIPRM, combined intra- and 6-mm peritumoral radiomics model; CM, clinical-radiological model; NM, nomogram model.

DeLong test showed that there were statistically significant differences between the NM and the other four models in the training set (p<0.05), and the NM had better predictive performance (**Table 4**). The 10-fold cross-validation shows that the NM exhibits excellent stability (**Supplementary Figure S3**). The calibration curve shows that the trend of the predicted curve of the pCR status by the NM is basically consistent with the actual curve (**Figure 6**). DCA shows that the NM has the greatest clinical net benefit in predicting pCR status after NAC in breast cancer patients (**Figure 7**).

**Table 4:** DeLong test for the comparison of ROC curves between different models in the training and test sets.

Models	Group	Z statistic	P-value
CM vs IRM	Training set	0.580	P = 0.562
	Test set	0.018	P = 0.985
CM vs PRM	Training set	0.550	P = 0.583
	Test set	0.019	P = 0.984
CM vs CIPRM	Training set	1.745	P = 0.081
	Test set	0.839	P = 0.401
CM vs NM	Training set	4.355	P < 0.001
	Test set	2.506	P = 0.012
IRM vs PRM	Training set	0.004	P = 0.997
	Test set	0.041	P = 0.967
IRM vs CIPRM	Training set	1.229	P = 0.219
	Test set	1.088	P = 0.276
IRM vs NM	Training set	2.693	P = 0.007
	Test set	2.042	P = 0.041
PRM vs CIPRM	Training set	1.231	P = 0.218
	Test set	0.924	P = 0.356
PRM vs NM	Training set	2.691	P = 0.007
	Test set	1.813	P = 0.069
CIPRM vs NM	Training set	2.718	P = 0.006
	Test set	1.546	P = 0.122

CM, clinical-radiological model; IRM, intratumoral radiomics model; PRM, 6-mm peritumoral radiomics model; CIPRM, combined intra- and 6-mm peritumoral radiomics model; NM, nomogram model; ROC, receiver operating characteristic.

## Discussion

In this study, we discussed the value of intratumoral combined peritumoral radiomics model based on pretherapy DCE-MRI images for early prediction of pCR. On this basis, we added clinical-radiological factors to construct a NM. The NM has better predictive performance, and has the potential to become a tool for early prediction of the post-NAC pCR status of breast cancer patients, contributing to the clinical formulation of precise treatment strategies, and helping patients avoid unnecessary surgery or reduce the toxic side effects of chemotherapy drugs.

Several clinical-radiological factors were significantly correlated with pCR, including Ki-67, ER and BES, indicating their potential value in predicting pCR status. This study found that patients with high expression of Ki-67 were more likely to achieve pCR, which is consistent with Kim et al. (28). The high expression of Ki-67 indicates that tumor cells are proliferating more actively, and chemotherapy drugs have strong lethality to cells with active proliferation. Therefore, patients with high expression of Ki-67 are more sensitive to chemotherapy drugs. This study showed that ER negative is an independent predictor of pCR, mainly because ER negative patients are more aggressive, have higher proliferation index and malignancy degree, are more sensitive to chemotherapy. Therefore, ER negative patients are more likely to obtain pCR than ER positive patients (29).BES is a key factor affecting the prognosis of breast cancer, which may be related to lymphovascular invasion (LVI) (30). Peritumoral edema is the mildest form of edema, prepectoral edema often indicates significant LVI, and subcutaneous edema often indicates more extensive LVI. This study found that BES was an independent predictor of pCR, and the incidence of BES was negatively correlated with that of pCR. Patients with no edema were more likely to achieve pCR than patients with peritumoral edema, prepectoral edema, or subcutaneous edema. This may be because LVI will make the tumor develop a certain resistance to chemotherapy drugs, thus affecting the efficacy of NAC, resulting in difficult to achieve pCR (31).

The peritumoral region often contains important biological information and is closely related to the development, metastasis and prognosis of the tumor. Xu et al. (32) extracted the intratumoral and 4-mm peritumoral regions radiomics features from digital breast tomosynthesis (DBT) to predict the LVI status of breast cancer patients, and found that the combined intratumoral and peritumoral radiomics models produced higher AUC. Zhang et al. (33) extracted the 2-mm, 4-mm, 6-mm, 8-mm peritumoral regions radiomics features from DCE-MRI images to predict the molecular subtypes of invasive ductal carcinoma. It is found that the optimal peritumoral region size is different for different prediction tasks. In recent years, there have been more and more studies on the prediction of pCR after NAC in breast cancer patients (34–36), but there are few studies on the optimal peritumoral region size. In the existing studies to predict pCR, different scholars set the size of the peritumoral region differently. Li et al. set 10-mm around the tumor as the peritumoral region (37), while Braman et al. set 2.5-5 mm around the tumor as the peritumoral region (38). These studies did not explore the optimal peritumoral region size. Mao et al. (39) extracted the intratumoral and 5-mm,10-mm peritumoral regions radiomics features from contrast-enhanced spectral mammography (CESM) to predict the effect of the NAC of breast cancers. The results showed that 5-mm around the tumor was the optimal peritumoral region. The combined intratumoral and 5-mm peritumoral radiomics model achieved a maximum AUC of 0.85 (95% CI, 0.72-0.98) in the test set. In this study, the radiomics features of 2-mm, 4-mm, 6-mm, 8-mm peritumoral regions were extracted from pretherapy DCE-MRI images to predict the pCR status of breast cancer patients after NAC, and the results showed that the 6-mm around the tumor was the optimal peritumoral region, the maximum AUC of the combined intratumoral and 6-mm peritumoral radiomics model in the training set and test set were 0.851 and 0.840, respectively, showing high predictive performance. Compared with previous research results, the model established in our study significantly improves the prediction performance, which may be related to the size of the selected peritumoral region. The peritumoral region reflects stromal involvement, immune infiltration, and angiogenesis, which are critical for neoadjuvant chemotherapy response. A 6-mm region may optimally capture these dynamics, as smaller regions (2-4 mm) might miss extended stromal interactions, while larger regions (8-mm) could introduce noise from normal tissues. Different peritumoral region sizes contain different tumor microenvironments, so we should determine the optimal peritumoral region size according to the actual prediction task, so as to improve the prediction performance of the model and obtain more valuable information.

In this study, five kinds of machine learning algorithms were used to establish the radiomics model. Among these radiomics models, CIPRM has the best predictive performance. In the CIPRM, SVM showed the best and stable performance. The SVM is often used to solve classification problems, which can deal with high-dimensional data sets and nonlinear relations between variables well, and is not easy to overfit, and has strong robustness and generalization. The CIPRM was established by SVM algorithm, and 10 radiomics features were extracted. The radiomics features were extracted from images after wavelet transform account for 70.0%(7/10), which may be due to the existence of pCR-related fine features in images after wavelet transform, whose details and complexity are higher than those of original images. Among the 10 radiomics features, there are 8 texture features and 2 first-order statistics. Texture features accounted for 80.0% (8/10), including 4 GLCM, 2 GLDM, 1 NGTDM and 1 GLSZM. Texture features can reflect the heterogeneity and complexity of breast cancer, and can indirectly predict the invasion ability, malignancy degree and prognosis of the tumor. The 10 radiomics features include 5 intratumoral and 5 peritumoral features. The coefficient of intratumoral feature (wavelet-LHH_gldm_LargeDependenceLowGrayLevelEmphasis) was the largest and positively correlated with the pCR rate, which was consistent with the results of Mao et al. (39). The coefficient of peritumoral feature (exponential_firstorder_Kurtosis) was second, which also reflects the importance of peritumoral radiomics features and can provide more supplementary information about the tumor microenvironment.

This study finally constructed five models for predicting pCR status after NAC in breast cancer patients, including IRM, 6-mm PRM, CIPRM, CM and NM. Among the five models, the NM has the best predictive performance. The AUC, sensitivity, specificity and accuracy of the NM was 0.911, 0.848, 0.831 and 0.826 in the training set, respectively, and 0.897, 0.893, 0.784 and 0.815 in the test set, respectively, which was superior to the single CM and three radiomics models. The CIPRM based on intratumoral and 6-mm peritumoral regions also showed better predictive performance, with the training set and test set AUC of 0.851 and 0.840, respectively, which was superior to the IRM, 6-mm PRM and CM. Different from Mao et al. (39), this study was based on DCE-MRI radiomics and clinical-radiological features to build a NM, which further improved the predictive performance of pCR. The nomogram shows that the CIPRM Rad-score has the greatest weight in the construction of the model, which reflects the importance of radiomics and can dig out more pathophysiological information of tumors.

This study has some limitations. First, it was a retrospective, single-center study that lacked external validation. The limited sample size raises concerns about potential overfitting risks. In future work, we will adopt multi-center and large-sample data to validate the model in order to further mitigate overfitting risks. Secondly, the evaluation of MRI radiological features may be influenced by the subjective factors of the radiologist. Finally, this study only constructed the model based on five machine learning algorithms. In our follow-up study, we plan to evaluate several deep learning architectures including: artificial neural networks (ANN), 3D convolutional neural networks (3D-CNN) for volumetric feature extraction, Hybrid radiomics-deep learning fusion models, Vision transformers (ViT) adapted for DCE-MRI analysis, etc. We will compare the performance between the NM and the deep learning-based model. In the future, we will utilize some XAI techniques such as Shapley Additive explanations (SHAP) or Local interpretable model-agnostic explanations (LIME) to enhance the explainability of both the machine learning- and deep learning-based models, thereby improving the clinical applicability of the models.

In summary, the NM established by combining intratumoral and peritumoral radiomics features and clinical-radiological factors has high performance, and can predict the pCR status of breast cancer patients after NAC in an early stage, providing an important information for clinical selection of appropriate treatment plan, timely adjustment of treatment strategy and prognosis assessment.

## Data Availability

The original contributions presented in the study are included in the article/**Supplementary Material**. Further inquiries can be directed to the corresponding authors.
