# Supplementary material for: Intra- and peritumoral radiomics nomogram based on DCE-MRI for the early prediction of pathological complete response to neoadjuvant chemotherapy in breast cancer
Source: Front Oncol. 2025 Jun 4;15:1561599. doi: 10.3389/fonc.2025.1561599 (PMC12174392; doi:10.3389/fonc.2025.1561599)
Supplement: Supplementary file 1 [file DataSheet1.doc]

*Supplementary Material*

**1. Supplementary Tables**

Table S1. Breast MRI sequences and acquisition parameters.

| Sequence | TR (ms) | TE (ms) | FOV (mm) | Matrix | Slice Thickness (mm) | Slice Gap  (mm) | Flip  Angle | b value |
| --- | --- | --- | --- | --- | --- | --- | --- | --- |
| T1WI | 5.7 | 2.4 | 340×340 | 234×225 | 4.5 | 1 | 90 |  |
| T2WI | 5000 | 60 | 337×337 | 336×303 | 4.5 | 1 | 90 |  |
| DWI | 3000 | 59 | 406×406 | 188×186 | 4.5 | 1 | 90 | 800 |
| DCE | 4.6 | 2.2 | 355×355 | 324×271 | 2 | 0 | 10 |  |

TR, repetition time; TE, echo time; FOV, field of view.

**Table S2.** The comparison of the prediction performance of the different machine learning algorithms.

| Model | Group | AUC | | | | |
| --- | --- | --- | --- | --- | --- | --- |
|  |  | LR | RF | SVM | KNN | XGBoost |
| IRM | Training set | 0.762 | 0.756 | 0.795 | 0.759 | 0.750 |
|  | Test set | 0.731 | 0.687 | 0.776 | 0.636 | 0.655 |
| 2-mm PRM | Training set | 0.712 | 0.682 | 0.751 | 0.692 | 0.733 |
|  | Test set | 0.652 | 0.669 | 0.746 | 0.635 | 0.711 |
| 4-mm PRM | Training set | 0.751 | 0.717 | 0.784 | 0.700 | 0.780 |
|  | Test set | 0.745 | 0.619 | 0.707 | 0.602 | 0.769 |
| 6-mm PRM | Training set | 0.794 | 0.722 | 0.762 | 0.688 | 0.748 |
|  | Test set | 0.779 | 0.684 | 0.740 | 0.666 | 0.698 |
| 8-mm PRM | Training set | 0.724 | 0.715 | 0.742 | 0.667 | 0.737 |
|  | Test set | 0.692 | 0.699 | 0.685 | 0.630 | 0.662 |
| CIPRM | Training set | 0.816 | 0.755 | 0.851 | 0.797 | 0.821 |
|  | Test set | 0.792 | 0.704 | 0.840 | 0.724 | 0.815 |

IRM, intratumoral radiomics model; PRM, peritumoral radiomics model; CIPRM, combined intra- and 6-mm peritumoral radiomics model; LR, LASSO regression; RF, random forest; SVM, support vector machines; KNN, K-nearest neighbor; XGBoost, extreme gradient boosting; AUC, area under the curve.

**Table S3.** The radiomics features and their respective coefficients of the three radiomics models.

| Model | Image filters | Feature type | Features | Coefficient |
| --- | --- | --- | --- | --- |
| IRM (10) | Gradient | First order | Minimum | +1.513 |
|  | wavelet-LHH | Gldm | LargeDependenceHighGrayLevelEmphasis | -0.934 |
|  | wavelet-HHH | Glszm | LargeAreaLowGrayLevelEmphasis | +0.907 |
|  | wavelet-LHL | Glcm | ClusterShade | -0.725 |
|  | Wavelet-LLH | Glszm | GrayLevelNonUniformityNormalized | -0.535 |
|  | wavelet-HHH | Glrlm | ShortRunHighGrayLevelEmphasis | -0.518 |
|  | wavelet-HHH | Ngtdm | Complexity | +0.326 |
|  | wavelet-HHH | Glcm | MaximumProbability | +0.308 |
|  | Local Binary Pattern (3D) | First order | MeanAbsoluteDeviation | +0.216 |
|  | wavelet-LHH | First order | 90Percentile | -0.080 |
| 6-mm PRM (11) | Gradient | First order | Kurtosis | +1.335 |
|  | Wavelet-HLH | Glcm | Imc1 | -1.280 |
|  | Wavelet-HHH | Glcm | MCC | -0.993 |
|  | Wavelet-HHL | Glcm | Idmn | -0.942 |
|  | Wavelet-HLH | Gldm_ | LargeDependenceHighGrayLevelEmphasis | +0.869 |
|  | Wavelet-LLL | Glcm | Correlation | -0.743 |
|  | Local Binary Pattern (3D) | First order | 90Percentile | -0.696 |
|  | Wavelet-HLL | First order | Mean | +0.683 |
|  | Wavelet-LHH | Gldm | SmallDependenceHighGray LevelEmphasis | -0.633 |
|  | Laplacian of Gaussian | Glrlm | ShortRunLowGrayLevelEmphasis | +0.556 |
|  | Exponential | First order | Skewness | +0.528 |
| CIPRM (10) | Wavelet-LHH | Gldm | LargeDependenceLowGray LevelEmphasis | +2.339 |
|  | Exponential | First order | Kurtosis | +2.184 |
|  | Gradient | Glszm | SmallAreaLowGrayLevelEmphasis | +1.763 |
|  | Wavelet-HLL | Glcm | ClusterTendency | +1.751 |
|  | Wavelet-HHL | Glcm | Idmn | -1.520 |
|  | Wavelet-LHH | First order | Kurtosis | -1.439 |
|  | Wavelet-HHH | Glcm | MCC | +1.205 |
|  | Wavelet-HLH | Gldm | LargeDependenceHighGray LevelEmphasis | +1.112 |
|  | Logarithm | Glcm | Imc2 | +0.702 |
|  | Wavelet-LLH | Ngtdm | Busyness | -0.631 |

**2. Supplementary Figures**


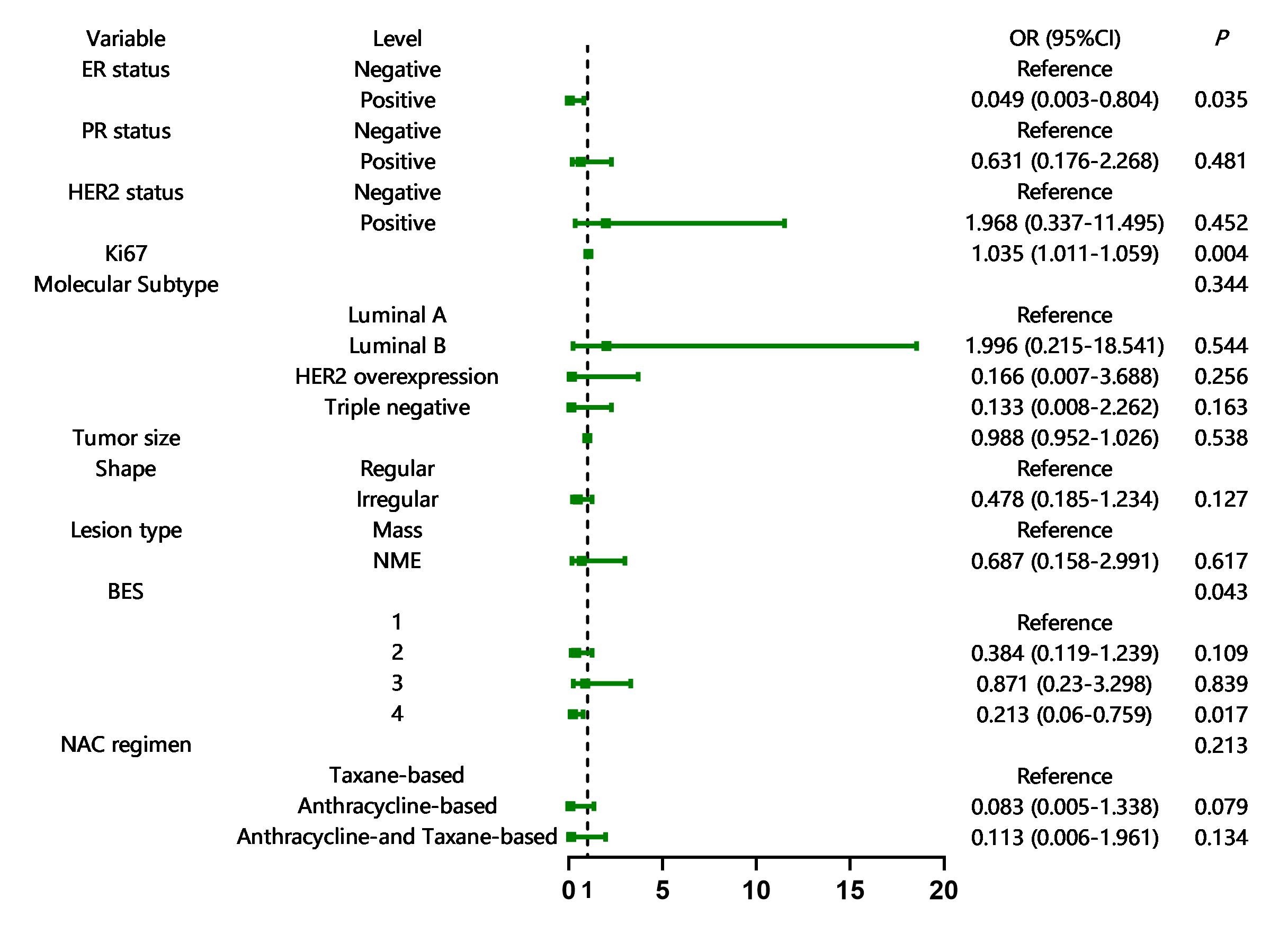


**Figure S1.** Forest plot of independent predictors of clinical-radiological features in the training set.

A

B







**Figure S2.** Confusion matrix of CIPRM in the training set (A) and test set (B).


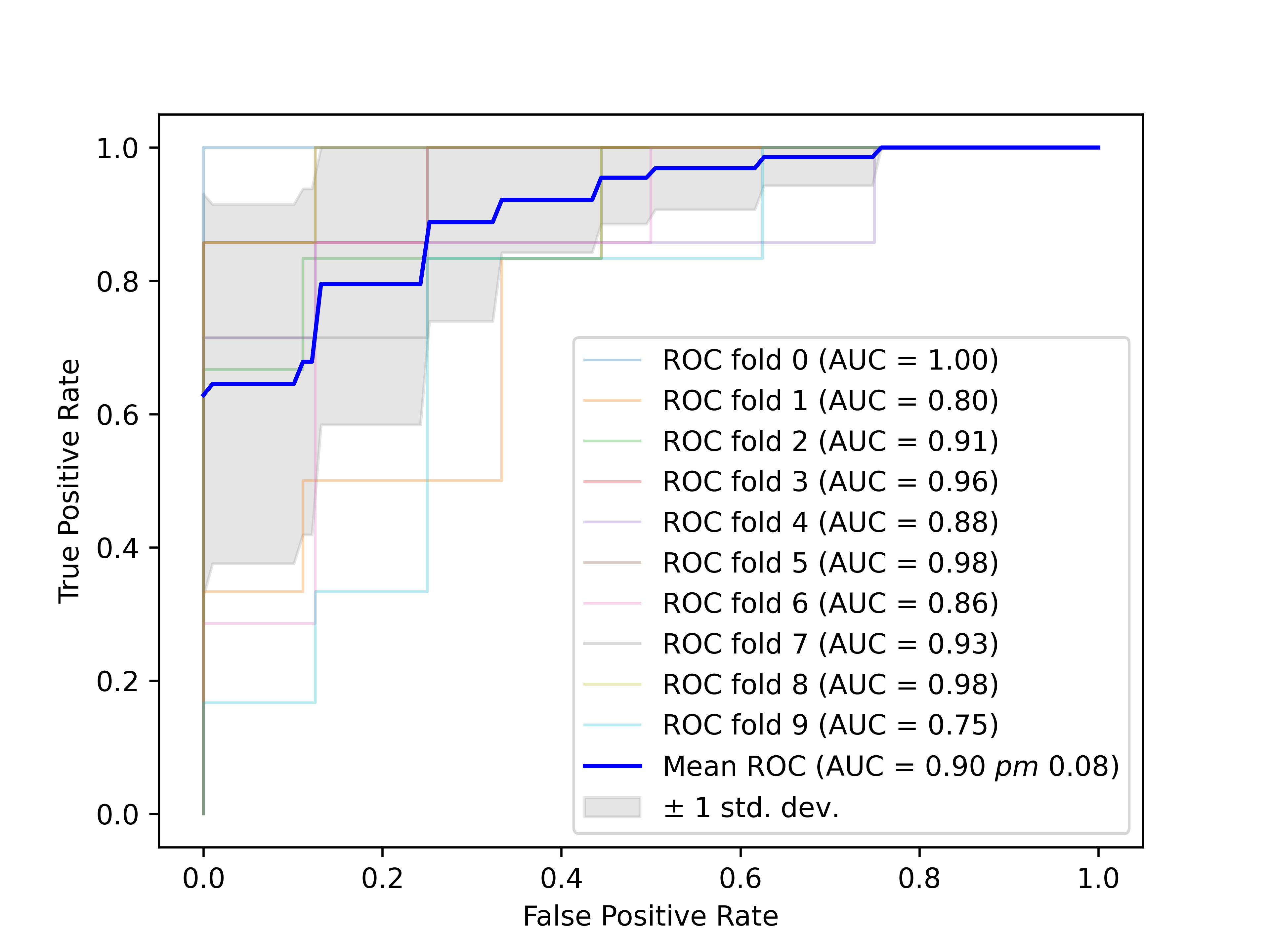


**Figure S3.** 10-fold cross-validation of the nomogram model.
